# An Oskar-Dependent Positive Feedback Loop Maintains the Polarity of the *Drosophila* Oocyte

**DOI:** 10.1016/j.cub.2006.12.044

**Published:** 2007-02-20

**Authors:** Vitaly Zimyanin, Nick Lowe, Daniel St Johnston

**Affiliations:** 1 Max Planck Institute of Molecular Cell Biology and Genetics, 108 Pfotenhauerstrasse, 01307 Dresden, Germany; 2 Wellcome Trust/Cancer Research UK, Gurdon Institute, University of Cambridge, Tennis Court Road, Cambridge CB2 1QN, United Kingdom

**Keywords:** CELLBIO, RNA, DEVBIO

## Abstract

The localization of *oskar* mRNA to the posterior of the *Drosophila* oocyte defines the site of assembly of the pole plasm, which contains the abdominal and germline determinants 1, 2, 3. *oskar* mRNA localization requires the polarization of the microtubule cytoskeleton, which depends on the recruitment of PAR-1 to the posterior cortex in response to a signal from the follicle cells, where it induces an enrichment of microtubule plus ends 4, 5, 6, 7. Here, we show that overexpressed *oskar* mRNA localizes to the middle of the oocyte, as well as the posterior. This ectopic localization depends on the premature translation of Oskar protein, which recruits PAR-1 and microtubule-plus-end markers to the oocyte center instead of the posterior pole, indicating that Oskar regulates the polarity of the cytoskeleton. Oskar also plays a role in the normal polarization of the oocyte; mutants that disrupt *oskar* mRNA localization or translation strongly reduce the posterior recruitment of microtubule plus ends. Thus, *oskar* mRNA localization is required to stabilize and amplify microtubule polarity, generating a positive feedback loop in which Oskar recruits PAR-1 to the posterior to increase the microtubule cytoskeleton's polarization, which in turn directs the localization of more *oskar* mRNA.

## Results and Discussion

### Overexpression of *oskar* mRNA Causes Premature Translation of Oskar Protein and an Assembly of the Pole Plasm at an Ectopic Site

During stage 9 of oogenesis, *oskar* mRNA localizes to the posterior of the *Drosophila* oocyte, where it nucleates the pole plasm, which contains the determinants that specify the germ cells and abdomen at the posterior of the embryo 1, 2, 3. This localization depends on the RNA-binding proteins Staufen and HRP48 and on the exon-junction-complex components Mago nashi, Y14, eIF4AIII, and Barentsz 8, 9, 10, 11, 12, 13, 14. In addition, the posterior accumulation of *oskar* mRNA requires microtubules and the plus-end-directed microtubule motor protein, kinesin, suggesting that it is transported by kinesin toward microtubule plus ends at the posterior pole 4, 15.

**Figure 1:**
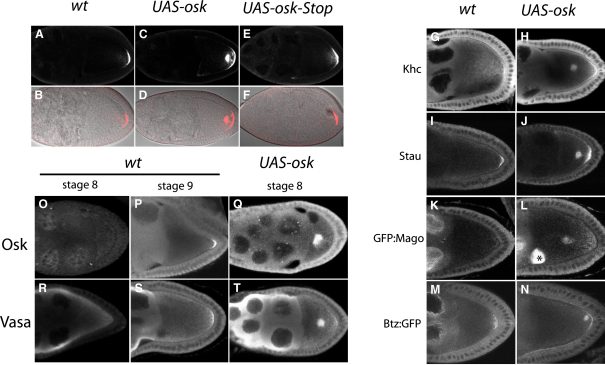
Overexpression of *oskar* mRNA Causes Its Mislocalization and Premature Translation in the Middle of the Oocyte (A–F) Fluorescence in situ hybridizations to *oskar* mRNA in wild-type (A and B), *UAS-osk* (C and D) and *UAS-osk-Stop* (E and F) stage 9 oocytes ([B], [D], and [F] were obtained by overlaying confocal pseudo differential-interference-contrast images with the in situ stainings [red channel] from the top panel [A, C, and E]). Overexpressed *oskar* mRNA is localized to both the posterior pole and an ectopic site in the middle of the *UAS-osk* oocytes. In flies overexpressing an untranslatable version of *oskar* mRNA (*UAS-osk-Stop*), the incidence of the ectopic localization is reduced, and most egg chambers show a wild-type pattern of localization (E and F). (G–N) Immunostainings for the Kinesin heavy chain (G and H), Stau (I and J), and localization of GFP-tagged variants of Mago nashi (K and L) and Barentsz (M and N) in wild-type (G, I, K, and M) and *UAS-osk* egg chambers (H, J, L, and N). All these components of the *oskar* mRNA localization complex are mislocalized to the center of the oocyte with overexpressed *oskar* mRNA. The asterisk in (L) indicates the strong accumulation of Mago:GFP in the oocyte nucleus. (O–Q) Immunostainings for Oskar protein. In wild-type egg chambers, Oskar protein is not expressed at stage 8 (O) and is first translated at the posterior pole of the oocyte at stage 9 (P). In *UAS-osk* oocytes, Oskar protein is translated in the middle of the oocyte at stage 8 (Q). (R–T) Immunostainings for Vasa protein. Oskar recruits Vasa protein to the posterior of the oocyte at stage 9 in wild-type egg chambers (R and S). In *UAS-osk* oocytes, the ectopic Oskar in the middle of the oocyte recruits Vasa at stage 8 (T).

As part of a project to visualize the transport of *oskar* mRNA, we used the *UAS-*GAL4 expression system to overexpress *oskar* mRNA in the female germline [16]. Although increased amounts of *oskar* mRNA are localized to the oocyte posterior in these *UAS-osk* females, a significant proportion of the mRNA is found in an ectopic dot in the middle of the oocyte in all stage 9 egg chambers (62/62) and more than half of those at stage 10A (80/150) (Figures 1A–1D).

The mislocalization of *oskar* mRNA to the center of the oocyte is reminiscent of the localization of *oskar* mRNA at stage 8 [17], suggesting that excess *oskar* mRNA saturates the transport pathway, causing a delay in its translocation to the posterior pole. We therefore examined the localization of other components of the *oskar* mRNA localization complex that are required for its transport to the posterior. Barentsz, Mago nashi, Staufen, and the Kinesin heavy chain all localize to the ectopic dot in the middle of the *oskar*-overexpressing oocytes, as well as to the posterior pole (Figures 1G–1N). Thus, none of these components appears to be limiting for transport to the posterior.

*oskar* mRNA is normally translationally repressed until it reaches the posterior pole, but overexpression of the *oskar* 3′ untranslated region (UTR) causes its premature translation [18]. We therefore examined whether this was also the case in the egg chambers overexpressing full-length *oskar* RNA. Oskar protein starts to accumulate at the posterior pole of wild-type egg chambers at stage 9 (Figures 1O and 1P). In UAS-*osk* egg chambers, robust Oskar expression is already visible in the ectopic dot at stage 8 (Figure 1Q). Thus, overexpressed *oskar* mRNA is not translationally repressed, even though it has not reached the posterior. Furthermore, the ectopic Oskar protein recruits Vasa, indicating that the premature translation of Oskar initiates the formation of the pole plasm in the middle of the oocyte (Figures 1R–1T).

To test whether Oskar protein is required for the mislocalization of *oskar* mRNA, we generated a second construct, UAS-*oskar*-Stop, in which we introduced a stop codon at position K178, which is equivalent to the mutation in the *oskar* protein-null allele *oskar*^54^ (Figures 1E and 1F). When *oskar*-STOP mRNA is overexpressed, it rarely localizes to the middle of the oocyte (13/79 at stage 9; 11/96 at stage 10A), although it is overexpressed at similar levels to the wild-type UAS-*oskar* mRNA (Figure S1E in the Supplemental Data online). Thus, the formation of the ectopic dot seems to be mainly caused by Oskar protein, rather than saturation of the localization pathway. The expression of the UAS-*oskar*-STOP construct induces the premature translation of endogenous *oskar* mRNA (see below), and this may account for the low penetrance of *oskar* mRNA mislocalization to the middle of these oocytes.

### UAS-*oskar*-STOP Activates Endogenous *oskar* Translation

Whereas *oskar* mutants block abdomen formation, extra copies of *oskar* cause the opposite bicaudal phenotype, in which the head and thorax are replaced by a mirror-image copy of the abdomen [19]. Although *UAS-osk* females produce some bicaudal embryos, the vast majority show a more extreme phenotype, in which the gut and terminal structures are expanded at the expense of the cuticle (Figures S1A–S1D, Table S1). This is most probably because *oskar* mRNA is very highly expressed in *UAS-osk* ovaries (equivalent to at least ten copies of *oskar*; see Figure S1E), leading to Oskar and Nanos overexpression, which causes an expansion of the expression domains of the terminal-gap genes, *tailless* and *huckebein* (data not shown) [20].

Surprisingly, UAS-*oskar*-STOP gives rise to embryonic phenotypes similar to those of UAS-*oskar*, even though it cannot produce functional Oskar protein (Figure S1D and Table S1). A weaker bicaudal phenotype has been observed when the *oskar-*3′UTR is overexpressed, leading to the proposal that this RNA activates the translation of endogenous *oskar* mRNA by titrating out translational repressors [18]. To test whether this is also the case for UAS-*oskar*-STOP, we expressed the transgene in females that were transheterozygous for two *oskar* protein-null mutations (*oskar*^54^/*oskar*^346^). The resulting embryos showed a typical posterior-group phenotype that was indistinguishable from *oskar*^54^/*oskar*^346^ alone (Table S1). Thus, UAS-*oskar*-STOP produces a bicaudal phenotype by activating the translation of endogenous wild-type *oskar* mRNA.

### Ectopic Pole Plasm Recruits Microtubule Plus Ends

At stage 7 of oogenesis, a signal from the posterior follicle cells induces a reorganization of the oocyte microtubule cytoskeleton, so that microtubules are then nucleated from the anterior and lateral cortex 21, 22, 23, 24, 25. The microtubule plus ends appear to localize to the middle of the oocyte during stages 7 and 8, as revealed by the localization of *oskar* mRNA, Staufen, and Kin:β-GAL (a marker for the plus ends of the microtubules) 4, 17, 26. This organization is only temporary, however, and plus-end markers start to accumulate at the posterior cortex at the beginning of stage 9, coincident with the onset of *oskar* mRNA localization to the posterior.

**Figure 2:**
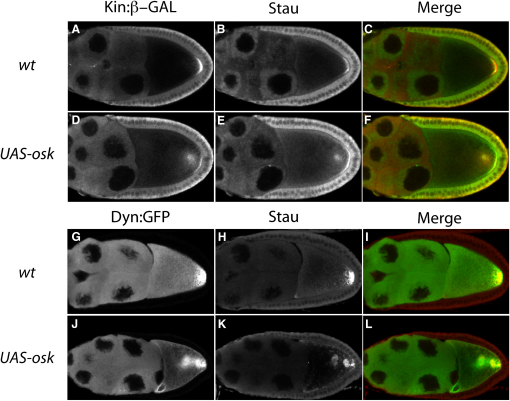
Markers for the Plus Ends of Microtubules Colocalize with Ectopic *oskar* mRNA (A–C) A wild-type egg chamber; Kin:β-GAL (A) localizes to the posterior pole of the oocyte at stage 9 and colocalizes with Staufen (B). (D–F) A *UAS-osk* egg chamber; Kin:β-GAL (D) and Staufen (E) are mislocalized to a dot in the center of the oocytes with overexpressed *oskar* mRNA, as well as to the posterior pole. (G–L) Dynamitin:GFP (G) fusion protein localizes to the posterior pole in wild-type egg chambers and colocalize with pole-plasm markers, such as Stau (H). In *UAS-osk* flies, Dynamitin:GFP (J) and Stau (K) are mislocalized to the ectopic site in the middle of the oocyte.

The mislocalization of *oskar* mRNA to the center of UAS-*oskar* oocytes resembles the polarity phenotype produced by *par-1* hypomorphic mutant combinations, in which some of the microtubule plus ends localize to the middle of the oocyte, rather than the posterior pole 5, 6. We therefore examined the localization of the plus-end marker Kinesin-β-GAL in UAS-*osk* oocytes. Kinesin-β-GAL always localizes to an ectopic site in the center of these oocytes (18/18), as well as to the posterior, and colocalizes with Staufen, a marker for *oskar* mRNA (Figures 2A–2F).

The dynein/dynactin motor complex provides another marker for the polarity of the microtubules because it is localized by kinesin to the posterior of the oocyte independently of *oskar* mRNA 17, 27, 28. This can be visualized with GFP-Dynamitin, a subunit of the Dynactin complex (Figures 2G–2I) [29]. GFP-Dynamitin is mislocalized with Staufen to the ectopic dot in the middle of almost all *oskar*-overexpressing oocytes (16/18) (Figures 2J and 2K). Thus, two markers for microtubule plus ends colocalize with ectopic Oskar in the middle of the oocyte.

The ectopic Oskar does not appear to interfere with the organization of the microtubule minus ends, or with minus-end-directed processes. The overall morphology of the microtubules is not affected when visualized with tubulin antibodies (not shown) or with a GFP-tagged version of the microtubule-binding protein Tau (Figures S2C and S2D). In addition, the oocyte nucleus always migrates normally to the dorsal-anterior corner of the oocyte, and *bcd* mRNA shows a wild-type localization to the anterior cortex (Figures S2A and S2B). Surprisingly, some *gurken* mRNA is often mislocalized to the posterior of UAS-*oskar* oocytes. It is not translated there, however, because Gurken protein is only found at the correct location adjacent to the oocyte nucleus (Figures S2E–S2I). This phenotype requires high levels of Oskar protein because *gurken* mRNA is not mislocalized in UAS-*oskar*-STOP oocytes. These results suggest that prematurely translated Oskar protein and/or downstream components of the pole plasm somehow sequester *gurken* mRNA and take it with them to the posterior pole.

### Oskar Is Required for the Normal Posterior Recruitment of Microtubule Plus Ends

The results above indicate that Oskar protein recruits microtubule plus ends when overexpressed, raising the possibility that Oskar normally plays a role in the recruitment of plus ends to the posterior in wild-type oocytes. To test this hypothesis, we re-examined the organization of the microtubule cytoskeleton in the absence of Oskar protein and in mutants that affect *oskar* mRNA localization and translation.

**Figure 3:**
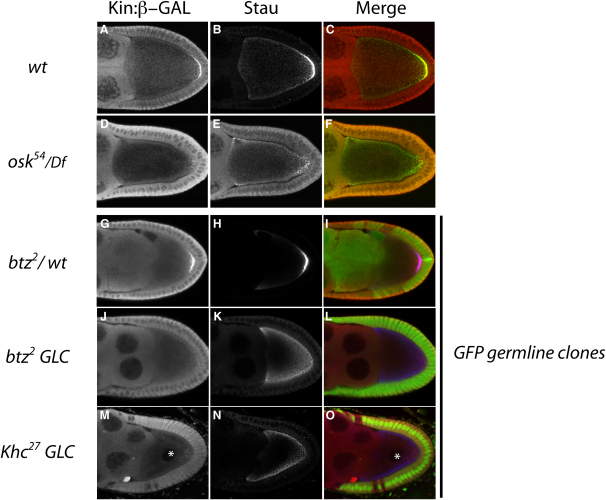
Oskar Is Required for the Efficient Recruitment of the Microtubule Plus Ends to the Posterior Pole (A–C) Wild-type: the plus-end marker, Kin:β-GAL, localizes to the posterior pole of the oocyte at stage 9 ([A], red in [C]) and colocalizes with Staufen ([B], green in [C]). (D–E) *osk^54^/Df*: The posterior localization of Kin:β-GAL ([D], red in [F]) and Staufen ([E], green in [F]) is strongly reduced in an *oskar* protein-null mutant. (G–I) A nonrecombinant *btz*^2^/+ egg chamber from a germline clone experiment, showing a robust localization of Kin:β-GAL ([G], red in [I]) and Staufen ([H], blue in [I]) to the posterior pole. GFP staining is shown in green in the merged image (I) and marks the nonrecombinant wild-type cells. The signal in the nurse cells indicates that this is not a germline clone. (J–L) A *btz*^2^ germline clone marked by the loss of GFP (green in [L]) from the same experiment as in (G)–(I). Kin:β-GAL (J) is barely detectable at the posterior of the oocyte, whereas Staufen (K) is not localized to the posterior pole, and accumulates at the anterior of the oocyte and diffusely around the oocyte cortex, consistent with the complete absence of *oskar* mRNA localization to the posterior pole in this mutant. (M–O) A *Khc*^27^ germline clone stained as above. Kin:β-GAL (M) is only very weakly localized to the posterior, whereas Staufen (O) fails to localize there and is mislocalized to the oocyte anterior-lateral cortex, where *oskar* mRNA is detected in *Khc*^27^ mutants. Also note the misplacement of the oocyte nucleus, labeled with the asterisk; this misplacement reflects the requirement for Kinesin in nuclear anchoring 28, 29, 38.

The plus-end marker Kin:β-GAL localizes to a tight posterior crescent in most wild-type stage 9 oocytes (88%, n = 58), with 12% displaying a weaker enrichment at the posterior pole (Figure 3A, Table S2). In an *oskar* protein null, however, only 16% of the oocytes show a robust posterior localization of Kin:β-GAL, whereas 72% show a weak posterior enrichment, and 12% show no localization to the posterior at all (Figure 3D). We also examined germline clones of *btz^2^* and *Khc^27^*, which completely disrupt *oskar* mRNA localization 12, 15. In these cases, the distribution of Kin:β-GAL in the mutant egg chambers can be directly compared with its localization in the heterozygous egg chambers from the same ovaries, which are stained under identical conditions. In each case, the mutant egg chambers show a highly penetrant reduction or loss of the posterior localization of Kin:β-GAL compared to the nonmutant controls (Figures 3G–3O, Table S2). These results are in good agreement with those of Brendza et al, who noted that Kin:β-GAL is not detectably localized in a significant fraction of *Khc* mutant oocytes [15]. Thus, the posterior recruitment of plus ends is strongly impaired when Oskar protein is absent from the posterior pole.

### Oskar May Reinforce Oocyte Polarity by Recruiting Par-1

The mechanism that recruits microtubule plus ends to the posterior pole at stage 9 is not well understood, but depends on the function of the conserved polarity kinase PAR-1 5, 6. In contrast to the wild-type, microtubules are nucleated all around the oocyte cortex of *par-1* mutants, and the plus ends are focused at the center instead of the posterior pole. As a consequence, *oskar* mRNA is mislocalized to the middle of the oocyte, and the resulting embryos lack an abdomen and germline. PAR-1 plays a similar role in organizing the microtubule cytoskeleton in both *Drosophila* and mammalian epithelial cells, indicating that one of its major functions is in the establishment of polarized microtubule arrays 30, 31, 32.

**Figure 4:**
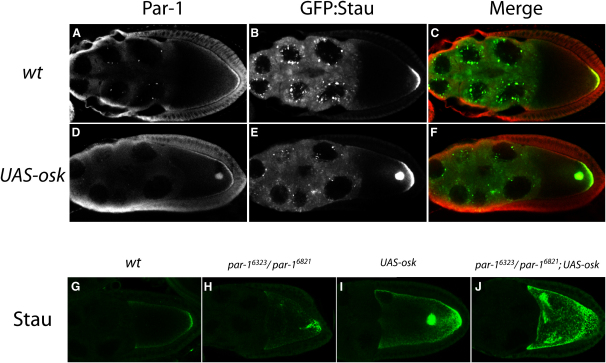
PAR-1 May Mediate Plus-End Recruitment by Oskar Protein (A–C) Wild-type: An immunostaining for PAR-1 ([A], red in [C]) shows its localization to the posterior with GFP-Staufen ([B], green in [C]). (D–F) UAS-*osk*: Overexpression of *oskar* mRNA leads to the recruitment of PAR-1 ([D], red in [F]) and GFP-Staufen ([E], green in [F]) to the middle of the oocyte. (G) An immunostaining for Stau protein shows its localization to the posterior pole of a wild-type oocyte. (H) In the hypomorphic mutant combination, *par-1^6323^/ par-1^6821^*, some Staufen is occasionally mislocalized to the middle of the oocyte. (I) In *UAS-osk*/GAL4, the overexpression of *oskar* mRNA also induces mislocalization of Staufen to the oocyte center. (J) *par-1^6323^/ par-1^6821^*; *UAS-osk*: *oskar* mRNA overexpression strongly enhances the weak *par-1* phenotype, leading to the complete loss of Staufen localization to the posterior.

The PAR-1N1 short and long isoforms appear to play the major role in the polarization of the oocyte cytoskeleton because only these fully rescue the polarity defects of *par-1* hypomorphs, and GFP-tagged PAR-1N1S and L are recruited to the posterior cortex of the oocyte at stage 7, making them the earliest markers for polarization of the anterior-posterior axis [7]. This localization is more cortical than that of the pole plasm and is not disrupted in *staufen*, *mago nashi*, and *Kinesin heavy chain* mutants, indicating that it is independent of *oskar* mRNA localization. Antibody stainings fail to detect the cortical PAR-1, perhaps because it is of too low abundance, but recognize a second population of PAR-1, which colocalizes to the posterior with the pole plasm 5, 6. This localization is disrupted in *oskar* and *staufen* mutants, but not *vasa* mutants, suggesting that PAR-1 is recruited by Oskar protein. These results suggest that *oskar* overexpression may interfere with polarity by recruiting PAR-1 to the center of the oocyte. In support of this view, antibody stainings revealed a strong localization of PAR-1 to the dot in the middle of the oocyte in *UAS-osk*-overexpressing flies (100%, n = 16; Figures 4A–4F). GFP-PAR-1N1S does not colocalize with ectopic Oskar in the center of the oocyte, however, consistent with the previous observation that this isoform is recruited to the posterior cortex, but not the pole plasm [25].

We also tested whether UAS-*oskar* enhances the phenotype of a weak *par-1* mutant combination. Flies transheterozygous for the two hypomorphic *par-1* alleles, *par-1^6323^* and *par-1^6821^*, have mild polarity defects, in which *oskar* mRNA and Staufen protein are mislocalized to the center of the oocyte in 56% (n = 136) of egg chambers (Figures 4G–4J, Table S3). Despite this mislocalization, Stau is also visibly enriched at the posterior pole of the oocyte in the majority of cases (88%). In contrast, *par-1^6323^*/*par-1^6821^* oocytes that also overexpress *oskar* mRNA show a much stronger phenotype: Stau protein is visibly enriched at the posterior pole of the oocyte in only 27% (n = 37) of these egg chambers and is mislocalized all around the oocyte cortex in most cases (Figure 4J; Table S3). In addition, these flies do not lay any eggs because of these severe defects in oogenesis. Thus, *oskar* overexpression strongly enhances the phenotype of a weak *par-1* mutant combination, most probably because ectopic Oskar protein recruits some of the limited amount of PAR-1 away from the posterior to the oocyte center.

Although *oskar* mRNA localization has always been presumed to be downstream of the polarization of the oocyte microtubule cytoskeleton, here we show through both gain- and loss-of-function experiments that it also plays an active role in this process, through the recruitment of microtubule plus ends by Oskar protein. PAR-1 is a good candidate to be the effector of Oskar function in plus-end recruitment because it is required for the polarization of the microtubule cytoskeleton and is recruited by Oskar protein to the posterior of the oocyte. Furthermore, the polarity phenotype of *par-1* hypomorphs is strongly enhanced by *oskar* mRNA overexpression, suggesting that PAR-1 and Oskar function in the same pathway to regulate the microtubules.

On the basis of our results, we would like to suggest a revised model for how the polarity of the oocyte is established (Figure S3). The polarization of the oocyte is initiated by the posterior-follicle-cell signal, which induces the localization of the PAR-1N1 isoforms to the posterior cortex. This localized PAR-1 then recruits or stabilizes some microtubule plus ends at the posterior cortex, leading to the kinesin-dependent transport of a small amount of *oskar* mRNA from the center of the oocyte to the posterior pole. Once *oskar* mRNA reaches the posterior, its translational repression is relieved, and the resulting Oskar protein recruits another population of PAR-1 to the posterior. This PAR-1 can then recruit further microtubule plus ends, which in turn direct the posterior localization of more *oskar* mRNA. Thus, Oskar protein initiates a positive feedback loop that amplifies the polarization of the microtubule cytoskeleton, leading to an increase in the localization of its own mRNA. The microtubule polarity is not reinforced in *oskar* protein-null mutants or in mutants that disrupt the localization of translation of *oskar* mRNA, and this results in only a partial polarization of the microtubule cytoskeleton. On the other hand, *oskar* mRNA overexpression and premature translation in the middle of the oocyte initiates the positive feedback loop in the wrong place. As a consequence, PAR-1 recruits some of the microtubule plus ends to the center of the oocyte and away from the posterior pole. PAR-1 has also been shown to stabilize Oskar protein through direct phosphorylation [33]. This therefore generates a second positive feedback loop that further reinforces the posterior localization of PAR-1, Oskar protein, and microtubule plus ends.

Amplification through positive feedback loops appears to be an emerging theme in cell polarity. For example, the polarization of migrating neutrophils depends on the local enrichment of phosphatidylinositol 3,4,5 triphosphate (PtdInsP_3_) at the leading edge of the cell, and this is amplified by a Rho-dependent positive feedback loop, in which PtdInsP_3_ recruits PtdIns-3-OH kinase to the leading edge, which in turn generates more PtdInsP_3_ [34]. A similar mechanism operates during bud-site selection in *S. cerevisiae*. The bud site is defined by the localization of activated Cdc42-GTP, and this localization induces the assembly of actin cables that extend into the cytoplasm. This signal is then reinforced by the transport of more Cdc42-GTP along the actin cables to the bud site, where it can induce the assembly of further actin cables [35]. This mechanism is enhanced by a second feedback loop, in which Cdc42-GTP recruits the adaptor protein, Bem1, which binds Cdc24, which activates Cdc42 36, 37. Our results add a third example of the use of multiple feedback loops to reinforce an initially weak cell polarity, and they provide the first case where this amplification involves the microtubule cytoskeleton rather than actin.

## Supplemental Data

Document S1. Experimental Procedures, Three Figures, and Three Tables

## References

[1] Ephrussi A., Dickinson L.K., Lehmann R. (1991). *oskar* organizes the germ plasm and directs localization of the posterior determinant *nanos*. Cell.

[2] Kim-Ha J., Smith J.L., Macdonald P.M. (1991). *oskar* mRNA is localized to the posterior pole of the *Drosophila* oocyte. Cell.

[3] Ephrussi A., Lehmann R. (1992). Induction of germ cell formation by *oskar*. Nature.

[4] Clark I., Giniger E., Ruohola-Baker H., Jan L., Jan Y. (1994). Transient posterior localisation of a kinesin fusion protein reflects anteroposterior polarity of the *Drosophila* oocyte. Curr. Biol..

[5] Shulman J.M., Benton R., St. Johnston D. (2000). The *Drosophila* homolog of C. *elegans* PAR-1 organizes the oocyte cytoskeleton and directs *oskar* mRNA localisation to the posterior pole. Cell.

[6] Tomancak P., Piano F., Riechmann V., Gunsalus K.C., Kemphues K.J., Ephrussi A. (2000). A *Drosophila melanogaster* homologue of *Caenorhabditis elegans par-1* acts at an early step in embryonic-axis formation. Nat. Cell Biol..

[7] Doerflinger H., Benton R., Torres I.L., Zwart M.F., St Johnston D. (2006). *Drosophila* anterior-posterior polarity requires actin-dependent PAR-1 recruitment to the oocyte posterior. Curr. Biol..

[8] St Johnston D., Beuchle D., Nüsslein-Volhard C. (1991). *Staufen*, a gene required to localize maternal RNAs in the Drosophila egg. Cell.

[9] Newmark P.A., Boswell R.E. (1994). The *mago nashi* locus encodes an essential product required for germ plasm assembly in *Drosophila*. Development.

[10] Hachet O., Ephrussi A. (2001). *Drosophila* Y14 shuttles to the posterior of the oocyte and is required for *oskar* mRNA transport. Curr. Biol..

[11] Mohr S.E., Dillon S.T., Boswell R.E. (2001). The RNA-binding protein Tsunagi interacts with Mago Nashi to establish polarity and localize *oskar* mRNA during *Drosophila* oogenesis. Genes Dev..

[12] van Eeden F.J.M., Palacios I.M., Petronczki M., Weston M.J.D., St Johnston D. (2001). Barentsz is essential for the posterior localization of *oskar* mRNA and colocalizes with it to the posterior. J. Cell Biol..

[13] Palacios I.M., Gatfield D., St Johnston D., Izaurralde E. (2004). An eIF4AIII-containing complex required for mRNA localization and nonsense-mediated mRNA decay. Nature.

[14] Huynh J.R., Munro T.P., Smith-Litiere K., Lepesant J.A., St Johnston D. (2004). The Drosophila hnRNPA/B homolog, Hrp48, is specifically required for a distinct step in osk mRNA localization. Dev. Cell.

[15] Brendza R.P., Serbus L.R., Duffy J.B., Saxton W.M. (2000). A function for kinesin I in the posterior transport of *oskar* mRNA and Staufen protein. Science.

[16] Brand A.H., Perrimon N. (1993). Targeted gene expression as a means of altering cell fates and generating dominant phenotypes. Development.

[17] Palacios I.M., St Johnston D. (2002). *Kinesin light chain*-independent function of the *Kinesin heavy chain* in cytoplasmic streaming, and posterior localization in the *Drosophila* oocyte. Development.

[18] Filardo P., Ephrussi A. (2003). Bruno regulates gurken during *Drosophila* oogenesis. Mech. Dev..

[19] Smith J.L., Wilson J.E., Macdonald P.M. (1992). Overexpression of *oskar* directs ectopic activation of *nanos* and presumptive pole cell formation in Drosophila embryos. Cell.

[20] Cinnamon E., Gur-Wahnon D., Helman A., St Johnston D., Jimenez G., Paroush Z. (2004). Capicua integrates input from two maternal systems in Drosophila terminal patterning. EMBO J..

[21] Theurkauf W.E., Smiley S., Wong M.L., Alberts B.M. (1992). Reorganization of the cytoskeleton during *Drosophila* oogenesis: Implications for axis specification and intercellular transport. Development.

[22] González-Reyes A., Elliott H., St Johnston D. (1995). Polarization of both major body axes in *Drosophila* by *gurken-torpedo* signalling. Nature.

[23] Roth S., Neuman-Silberberg F.S., Barcelo G., Schüpbach T. (1995). *cornichon* and the EGF receptor signaling process are necessary for both anterior-posterior and dorsal-ventral pattern formation in *Drosophila*. Cell.

[24] Clark I., Jan L.Y., Jan Y.N. (1997). Reciprocal localization of Nod and kinesin fusion proteins indicates microtubule polarity in the *Drosophila* oocyte, epithelium, neuron and muscle. Development.

[25] Cha B., Koppetsch B.S., Theurkauf W.E. (2001). In Vivo Analysis of Drosophila *bicoid* mRNA Localization Reveals a Novel Microtubule-Dependent Axis Specification Pathway. Cell.

[26] Cha B.J., Serbus L.R., Koppetsch B.S., Theurkauf W.E. (2002). Kinesin I-dependent cortical exclusion restricts pole plasm to the oocyte posterior. Nat. Cell Biol..

[27] Li M.-g., McGrail M., Serr M., Hays T.H. (1994). *Drosophila* cytoplasmic dynein, a microtubule motor that is asymmetrically localized in the oocyte. J. Cell Biol..

[28] Brendza R., Serbus L., Saxton W., Duffy J. (2002). Posterior localization of Dynein and dorsal-ventral axis formation depend on Kinesin in *Drosophila* oocytes. Curr. Biol..

[29] Januschke J., Gervais L., Dass S., Kaltschmidt J.A., Lopez-Schier H., St Johnston D., Brand A.H., Roth S., Guichet A. (2002). Polar transport in the Drosophila oocyte requires Dynein and Kinesin I cooperation. Curr. Biol..

[30] Drewes G., Ebneth A., Preuss U., Mandelkow E.M., Mandelkow E. (1997). MARK, a novel family of protein kinases that phosphorylate microtubule- associated proteins and trigger microtubule disruption. Cell.

[31] Doerflinger H., Benton R., Shulman J.M., St Johnston D. (2003). The role of PAR-1 in regulating the polarised microtubule cytoskeleton in the Drosophila follicular epithelium. Development.

[32] Cohen D., Brennwald P.J., Rodriguez-Boulan E., Musch A. (2004). Mammalian PAR-1 determines epithelial lumen polarity by organizing the microtubule cytoskeleton. J. Cell Biol..

[33] Riechmann V., Gutierrez G.J., Filardo P., Nebreda A.R., Ephrussi A. (2002). Par-1 regulates stability of the posterior determinant Oskar by phosphorylation. Nat. Cell Biol..

[34] Weiner O.D., Neilsen P.O., Prestwich G.D., Kirschner M.W., Cantley L.C., Bourne H.R. (2002). A PtdInsP(3)- and Rho GTPase-mediated positive feedback loop regulates neutrophil polarity. Nat. Cell Biol..

[35] Wedlich-Soldner R., Altschuler S., Wu L., Li R. (2003). Spontaneous cell polarization through actomyosin-based delivery of the Cdc42 GTPase. Science.

[36] Irazoqui J.E., Gladfelter A.S., Lew D.J. (2003). Scaffold-mediated symmetry breaking by Cdc42p. Nat. Cell Biol..

[37] Wedlich-Soldner R., Wai S.C., Schmidt T., Li R. (2004). Robust cell polarity is a dynamic state established by coupling transport and GTPase signaling. J. Cell Biol..

[38] Duncan J.E., Warrior R. (2002). The cytoplasmic Dynein and Kinesin motors have interdependent roles in patterning the *Drosophila* oocyte. Curr. Biol..

