# Supplementary material for: An Oskar-Dependent Positive Feedback Loop Maintains the Polarity of the Drosophila Oocyte
Source: Curr Biol. 2007 Feb 20;17(4):353–9. doi: 10.1016/j.cub.2006.12.044 (PMC1885951; doi:10.1016/j.cub.2006.12.044)
Supplement: Document S1. Experimental Procedures, Three Figures, and Three Tables [file mmc1.pdf]

Supplemental Data

An Oskar-Dependent Positive Feedback Loop Maintains the Polarity of the *Drosophila* Oocyte

Vitaly Zimyanin, Nick Lowe, and Daniel St Johnston

Supplemental Experimental Procedures

Generation of *UAS-osk* and *UAS-osk-Stop* Lines

A BamH1 and Hinc2 genomic DNA fragment containing the *osk* gene was cloned from the I4.1 cosmid (kindly provided by A. Ephrussi) into the BamH1 and Pst1 sites of the p*UAS*-p transformation vector [S1]. For making a nonsense version of the same construct, in vitro mutagenesis was used to change A860 to T, which introduces a premature stop codon in place of K178. The resultant transgenes were introduced into flies through standard germline transformation techniques.

Fly Strains and Germline Clones

oskar mRNA overexpression was performed with w^-*; P [*w^+*; mat-α4-tub-GAL4VP16 V32a]/CyO*, or w^-*; ;P [*w^+*; mat-α4-tub-GAL4VP16 V37]* as *GAL4* drivers (D.StJ. and J.-P. Vincent, unpublished data). Other strains used were as follows: *y,* w^-*, P [*w^+*; mat-tub-α4:GFP Staufen], [*ry^+*; hs:FLP]; Pr, Dr/TM6B, Tb* [S2]; w^- *;P[*w^+*, tub-Btz GFP-24A2] /TM6B* [S3]; w^- *;P[*w^+*, GFPMago17.1]/CyO* [S4]; osk^{54} and a deficiency that uncovers *osk, Df(3R)p*^{XT103} [S5]; w^-*, P[*w^+*, UASp-Tau:GFP]* [S4]; *par-1*^{6323}*, par-1*^{6821} [S6]; w^-*, P[KZ503, Kin:β-GAL], P[*ry^+*; hs:FLP]* [S7]; and w^-*; P [*w^+*, UAS-GFP-dDyn]/CyO* [S8].

Antibodies and Immunofluorescence

Ovary and embryo fixations and antibody stainings were performed according to standard procedures. In situ hybridizations were performed with RNA probes labeled with Digoxigenin-UTP (Roche), and anti-DIG secondary antibody coupled to Cy-3 (Jackson Immunoresearch).

Primary antibodies and their dilutions were as follows: polyclonal rabbit anti-Osk (1:2000) [S9], rabbit anti-Stau (1:1000) [S10], rabbit anti-Par1 [S11] (1:5000), rabbit anti-Btz [S3] (1:500–1:1000), rat anti-Vasa [S12] (1:1000), mouse anti-β-GAL (1:2000, Sigma), and rabbit anti-Khc (1:250, Cytoskeleton company).

Microtubules were stained with FITC-conjugated monoclonal α-tubulin antibody (Sigma) at a 1:250 dilution in PBST as described in [S13].

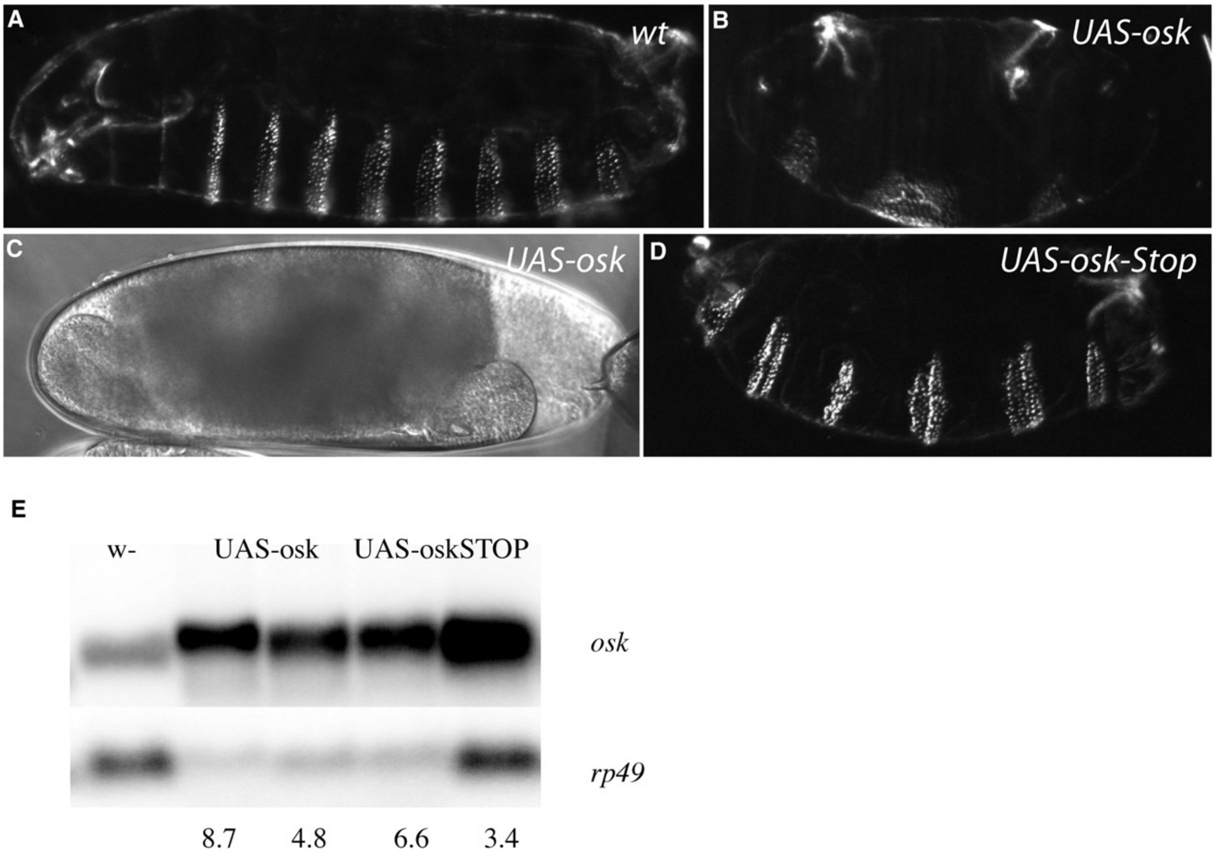

Figure S1. *oskar* mRNA Overexpression Causes Severe Defects in the Anterior-Posterior Patterning of the Embryo

(A–D) Cuticle preparations (A, B, and D) and a phase-contrast image (C) of embryos from wild-type (A), *UAS-osk* (B and C), and *UAS-osk-Stop* (D) mothers. Overexpression of *oskar* mRNA causes a bicaudal phenotype with a mirror-image duplication of posterior abdominal segments and of the telson (B). The majority of *UAS-osk* embryos develop an extreme bicaudal phenotype, in which embryos have a large expansion of the foregut and hindgut, which protrude from the ends of the embryo. These embryos still have duplicated telsons at each end (not seen in the phase contrast image), but lack all other cuticular structures (C). *UAS-osk-Stop* embryos display slightly weaker patterning defects, with fewer extreme bicaudals and more embryos with a symmetric bicaudal phenotype or a weak bicaudal phenotype, in which the head is replaced by a duplicated telson.

(E) Northern blot showing the levels of *oskar* mRNA in wild-type, Gal4/*UAS-osk*, and Gal4/*UAS-osk-STOP* ovaries. The blot was reprobed with rp49 as a loading control (lower panel). The relative overexpression of *oskar* mRNA compared to wild-type ovaries is shown beneath each lane.

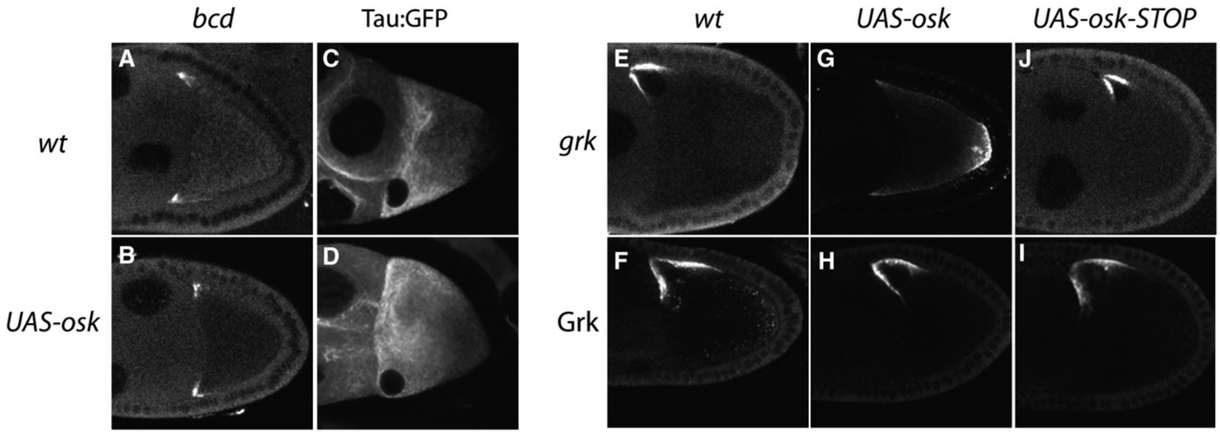

Figure S2. *oskar* mRNA Overexpression Does Not Affect Minus-End-Directed Processes

(A and B) Fluorescent in situ hybridization for *bcd* mRNA in wild-type (A) and *UAS-osk* flies (B). *bcd* mRNA localization to the anterior pole is not affected by *oskar* mRNA overexpression (B).

(C and D) Tau:GFP labeling of microtubules in living wild-type (C) and *UAS-osk* oocytes (D). The overall organization of the microtubule cytoskeleton appears normal in *UAS-osk* egg chambers (D).

(E–I) *grk* mRNA is localized to the anterior-dorsal corner of the oocyte in wild-type (E) and in *UAS-osk-STOP* (J) egg chambers, but is often partially mislocalized to the posterior of *UAS-osk* oocytes (G). Grk protein, however, is only translated at its normal position at the anterior-dorsal corner of the oocyte in wild-type (F), *UAS-osk* (H), and *UAS-osk-STOP* (I) egg chambers.

Texas-Red-, FITC-, and Cy5-conjugated secondary antibodies were used at 1/100 (Molecular Probes). Rhodamine- or TRITC-conjugated Phalloidin was used to visualize the actin cytoskeleton (Molecular Probes).

Northern Blotting

RNA was prepared from 30–50 fly ovaries dissected in RNA-Later (Ambion) and purified with RNA Easy minikit (Qiagen). After electrophoresis with standard methods, RNA was blotted onto Zeta-Probe membrane (Bio-Rad) and hybridized sequentially with probes for the *oskar* coding sequence and the ribosomal protein rp49. Probes were labeled with [α-^{32}P] dCTP with the DECA Prime labeling system (Ambion). After exposure on a phosphorimager plate, blots were scanned and quantitated on a FLA-5000 scanner and Image Gauge 4.0 (FujiFilm).

Supplemental References

S1. Rorth, P. (1998). Gal4 in the Drosophila female germline. Mech. Dev. *78*, 113–118.

S2. Martin, S.G., Leclerc, V., Smith-Litiere, K., and St Johnston, D. (2003). The identification of novel genes required for *Drosophila* anteroposterior axis formation in a germline clone screen using GFP-Staufen. Development *130*, 4201–4215.

S3. van Eeden, F.J.M., Palacios, I.M., Petronczki, M., Weston, M.J.D., and St Johnston, D. (2001). Barentsz is essential for the posterior localization of *oskar* mRNA and colocalizes with it to the posterior. J. Cell Biol. *154*, 511–524.

S4. Micklem, D.R., Dasgupta, R., Elliott, H., Gergely, F., Davidson, C., Brand, A., González-Reyes, A., and St Johnston, D. (1997). The *mago nashi* gene is required for the polarisation of the oocyte and the formation of perpendicular axes in *Drosophila.* Curr. Biol. *7*, 468–478.

S5. Lehmann, R., and Nüsslein-Volhard, C. (1986). Abdominal segmentation, pole cell formation, and embryonic polarity require the localized activity of *oskar*, a maternal gene in Drosophila. Cell *47*, 141–152.

S6. Shulman, J.M., Benton, R., and St. Johnston, D. (2000). The *Drosophila* homolog of C. *elegans* PAR-1 organizes the oocyte cytoskeleton and directs *oskar* mRNA localisation to the posterior pole. Cell *101*, 1–20.

S7. Clark, I., Giniger, E., Ruohola-Baker, H., Jan, L., and Jan, Y. (1994). Transient posterior localisation of a kinesin fusion protein reflects anteroposterior polarity of the *Drosophila* oocyte. Curr. Biol. *4*, 289–300.

S8. Januschke, J., Gervais, L., Dass, S., Kaltschmidt, J.A., Lopez-Schier, H., St Johnston, D., Brand, A.H., Roth, S., and Guichet, A. (2002). Polar transport in the Drosophila oocyte requires Dynein and Kinesin I cooperation. Curr. Biol. *12*, 1971–1981.

S9. Vanzo, N.F., and Ephrussi, A. (2002). Oskar anchoring restricts pole plasm formation to the posterior of the *Drosophila* oocyte. Development *129*, 3705–3714.

S10. St Johnston, D., Beuchle, D., and Nüsslein-Volhard, C. (1991). *Staufen*, a gene required to localize maternal RNAs in the Drosophila egg. Cell *66*, 51–63.

S11. Sun, T.-Q., Lu, B., Feng, J.-J., Reinhard, C., Jan, Y.N., Fantl, W.J., and Williams, L.T. (2001). PAR-1 is a Dishevelled-associated kinase and a positive regulator of Wnt signalling. Nat. Cell Biol. *3*, 628–636.

S12. Lasko, P.F., and Ashburner, M. (1990). Posterior localization of *vasa* protein correlates with, but is not sufficient for, pole cell development. Genes Dev. *4*, 905–921.

S13. Cha, B., Koppetsch, B.S., and Theurkauf, W.E. (2001). In Vivo Analysis of Drosophila *bicoid* mRNA Localization Reveals a Novel Microtubule-Dependent Axis Specification Pathway. Cell *106*, 35–46.

S14. Cinnamon, E., Gur-Wahnon, D., Helman, A., St Johnston, D., Jimenez, G., and Paroush, Z. (2004). Capicua integrates input from two maternal systems in Drosophila terminal patterning. EMBO J. *23*, 4571–4582.

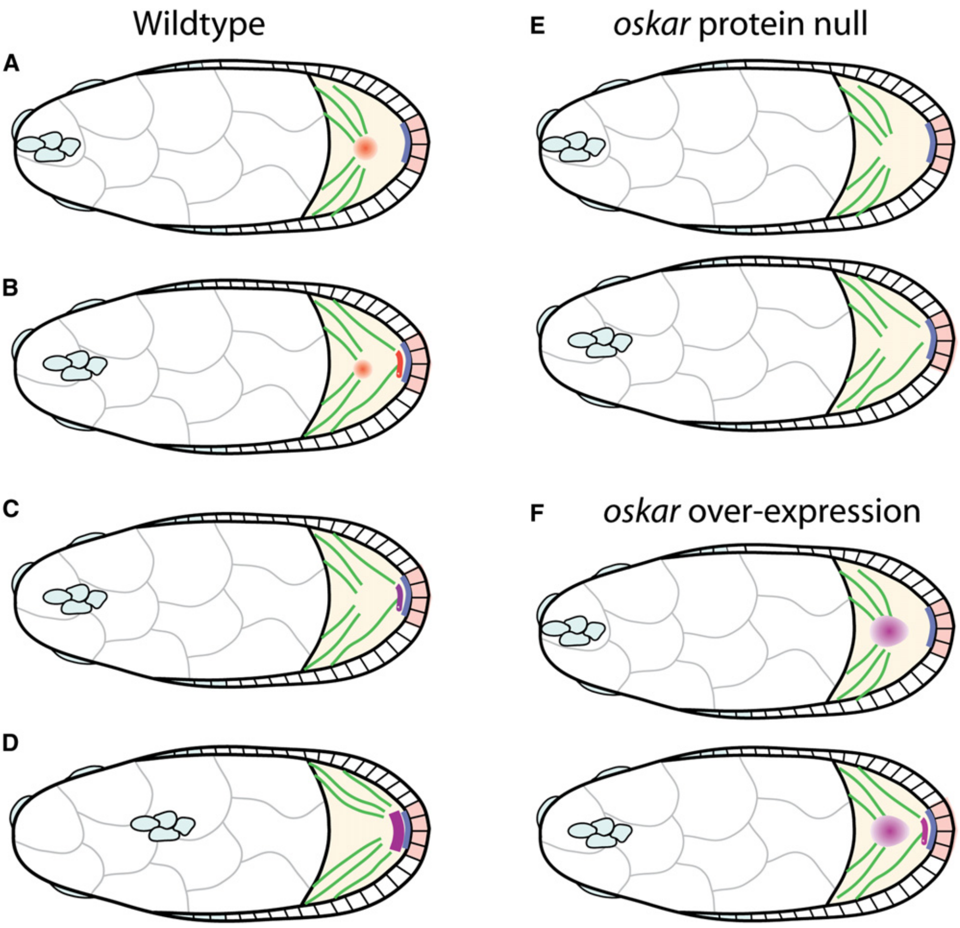

Figure S3. A Model for an Oskar-Dependent Positive Feedback Loop that Amplifies Oocyte Polarity

(A) By stage 8 of oogenesis, the microtubule cytoskeleton has partially repolarized in response to the signal from the posterior follicle cells. The microtubules (green) are nucleated from the anterior and lateral cortex, but their plus ends have not been recruited to posterior and direct the localization of *oskar* mRNA (red) to the middle of the oocyte. The follicle-cell signal recruits the N1 isoforms of PAR-1 (blue) to the posterior cortex of the oocyte.

(B) The posterior PAR-1 recruits some microtubule plus ends to the posterior, and this allows the localization of small amount of *oskar* mRNA to the posterior pole.

(C) *oskar* mRNA is translationally derepressed at the posterior, resulting in the synthesis of Oskar protein, which recruits a second population of PAR-1 to the posterior (shown in purple to indicate the association of PAR-1, Oskar protein, and *oskar* mRNA).

(D) The additional PAR-1 generates a positive feedback loop by stabilizing more microtubule plus ends at the posterior, leading to an increased posterior localization of *oskar* mRNA and protein, which in turn recruit more PAR-1.

(E) In an *oskar* protein null mutant, the second population of PAR-1 is not recruited to the posterior by Oskar protein, and the plus ends of the microtubules show only partial and unstable recruitment of to the posterior pole.

(F) Overexpression of *oskar* mRNA leads its premature translation in the middle of the oocyte. The resulting ectopic Oskar protein recruits PAR-1, which induces the mispositioning of some microtubule plus ends in the center of the oocyte.

Table S1. Embryonic Phenotypes Observed in *UAS-osk*, *UAS-osk-STOP*, and *UAS-osk3′UTR* Flies

		Patterning Defects, Head Structures Are Not Affected		Bicaudals			
	Wild-type	5–7 Denticle Belts	0–4 Denticle Belts	Asymmetric Bicaudals	Symmetric Bicaudals with Denticle Belts	Extreme Bicaudals, No Denticle Belts	Total
w^{-}	100%	—	—	—	—	—	39
GAL4 / TM3[a]	68%	32%	—	—	—	—	164
UASosk / GAL4[b]	0.5%	0.5%	—	1%	3%	95%	247
UASosk-Stop / GAL4[b]	17%	—	—	13%	41%	29%	328
osk^{54}/osk^{346}		0.4%	99.6%	—	—	—	225
UASosk-stop/ GAL4; osk^{54} *or* osk^{346}*/ +*	67%	27%	1%	4%	1%	—	92
UASosk-stop/ GAL4; osk^{54}/osk^{346}	—	—	100%	—	—	—	76

[a] *mat-tub-a4-GAL4VP16 V37* line used in this experiment has been reported to have nonspecific patterning defects [S14].
[b] Similar phenotypic classes were observed in two independent transgenic lines.

Table S2. Kin:β-GAL Localization in Osk Protein-Null Conditions and in btz^{2} and Khc^{27} Mutant GFP Germline Clones

	Wild-type	Reduced Localization to the Posterior	No Localization to the Posterior Pole	Total
w^{-}	88%	12%	—	40
osk^{54}/Df	16%	72%	12%	100
btz^{2}	25%	55%	20%	20
Khc^{27}	15%	62%	23%	13

Table S3. *oskar* mRNA Overexpression Enhances the Polarity Defects in Flies Heterozygous for Two Hypomorphic Alleles of *par-1*

	Wild-type Posterior Stau	Ectopic Stau Localization with Some Enrichment at the Posterior Pole	No Enrichment of Stau at the Posterior Pole	Total
w^{-}	100%	—	—	31
$par\text{-}1^{6323}/\ par\text{-}1^{6821}$	32%	56%	12%	136
UAS-osk /Gal4vp16	23%	77%	0%	52
$par\text{-}1^{6323}/\ par\text{-}1^{6821}$*; UAS-osk/Gal4vp16*	0%	27%	73%	37

The table gives the percentage of oocytes of each genotype that show a wild-type posterior localization of Staufen, localization of Staufen to the posterior and an ectopic dot, or no posterior localization with one or more ectopic sites of Staufen localization.
